# Termination of Longstanding Persistent Atrial Fibrillation Using a Novel Large Focal Lattice-Tip Pulsed Field Ablation Catheter: A Case Report

**DOI:** 10.7759/cureus.104019

**Published:** 2026-02-21

**Authors:** Havish S Kantheti, Ramya Natarajan, Aleem Mughal

**Affiliations:** 1 Internal Medicine, Baylor Scott & White Health, Fort Worth, USA; 2 Electrophysiology, Heart Center of North Texas, Fort Worth, USA

**Keywords:** 3d electrophysiology mapping, atrial fibrillation management, cardiac electrophysiology, electrophysiology, pulsed field ablation

## Abstract

Catheter ablation in longstanding persistent atrial fibrillation (AF) presents unique challenges, particularly in patients with large left atria and prior atrioventricular nodal dysfunction. While novel pulsed-field ablation (PFA) has been shown to be safer, no data yet show that a large focal lattice-tip catheter may be more effective. We present a 66-year-old man with symptomatic, longstanding, persistent AF and underlying sick sinus syndrome who underwent catheter ablation using Affera 3D mapping (Medtronic, Minneapolis, MN) and the Sphere 9 catheter (Medtronic, Inc, Mounds View, MN) to provide a combined pulsed field and radiofrequency approach. High-resolution electroanatomic mapping guided pulmonary vein isolation, which was extended to include posterior wall isolation, mitral isthmus ablation, and cavotricuspid isthmus ablation due to multiple arrhythmia foci. During delivery of PFA to complete posterior wall isolation, there was termination of AF to a normal sinus rhythm. This case highlights the utility of comprehensive lesion sets and advanced mapping technologies in achieving rhythm control in complex persistent AF.

## Introduction

Atrial fibrillation (AF) is the most common sustained cardiac arrhythmia and is associated with increased risks of stroke, heart failure, and healthcare utilization. Catheter ablation, centered on pulmonary vein isolation (PVI), is a cornerstone of rhythm control for symptomatic, drug-refractory AF [1,2]. However, outcomes are less favorable in longstanding persistent AF, where advanced atrial remodeling, left atrial enlargement, and nonpulmonary vein (non-PV) substrates often contribute to arrhythmia maintenance [3,4]. In this population, PVI alone is frequently insufficient, prompting investigation into adjunctive lesion sets such as posterior wall isolation and linear ablation.

Prior randomized trials, including Substrate and Trigger Ablation for Reduction (STAR)-AF II, did not demonstrate incremental benefit from empiric linear ablation or complex fractionated electrogram targeting beyond PVI [3]. These findings have shaped contemporary practice but were generated using earlier mapping technologies and thermal energy sources, which may limit their applicability to modern substrate-guided approaches. As a result, the role of additional lesion sets in longstanding persistent AF remains an area of active debate. Posterior wall isolation, in particular, has shown mixed results across studies, with ongoing investigation into its potential benefit in selected patients with persistent AF [5].

Pulsed field ablation (PFA) represents a nonthermal ablation modality that delivers high-voltage electrical pulses to induce irreversible electroporation of myocardial tissue while relatively sparing adjacent structures such as the esophagus and phrenic nerve [6,7]. Clinical studies have demonstrated favorable safety and efficacy profiles for PFA in PVI, with emerging experience in posterior wall and linear ablation [8,9]. However, most published clinical data to date involve single-shot or multielectrode PFA systems designed for circumferential lesion delivery.

In contrast, focal lattice-tip catheters enable high-resolution electroanatomic mapping and focal energy delivery, with the ability to toggle between pulsed field and radiofrequency (RF) energy within a single platform. This design may allow more tailored substrate modification in anatomically complex regions, such as the posterior left atrium or mitral isthmus, while preserving the tissue-selective advantages of PFA [10]. Despite growing adoption, clinical experience with lattice-tip focal PFA catheters in patients with longstanding persistent AF remains limited.

In this case report, we describe a patient with symptomatic, longstanding, persistent AF and advanced atrial remodeling who underwent catheter ablation using a focal lattice-tip catheter delivering both pulsed-field and RF energy. The case illustrates the feasibility of comprehensive lesion sets, including posterior wall and linear ablation, and raises the clinical question of whether focal lattice-tip PFA may facilitate effective engagement of non-PV substrates in selected patients with advanced atrial disease.

## Case presentation

A 66-year-old man with a history of nonobstructive coronary artery disease, hypertension, diabetes mellitus, and sick sinus syndrome presented with symptomatic, longstanding, persistent AF, defined as continuous AF for more than 12 months. Despite amiodarone therapy, he reported persistent symptoms and awareness of continuous AF with associated atrioventricular (AV) dyssynchrony.

Procedure

After informed consent, the patient underwent catheter ablation in the fasting state. A preprocedural transesophageal echocardiogram excluded left atrial appendage thrombus. Venous access was obtained via the right femoral vein under ultrasound guidance, and anticoagulation was maintained with an activated clotting time greater than 300 seconds throughout the procedure. A single transseptal puncture was performed with intracardiac echocardiographic (ICE) and fluoroscopic guidance.

**Figure 1:**
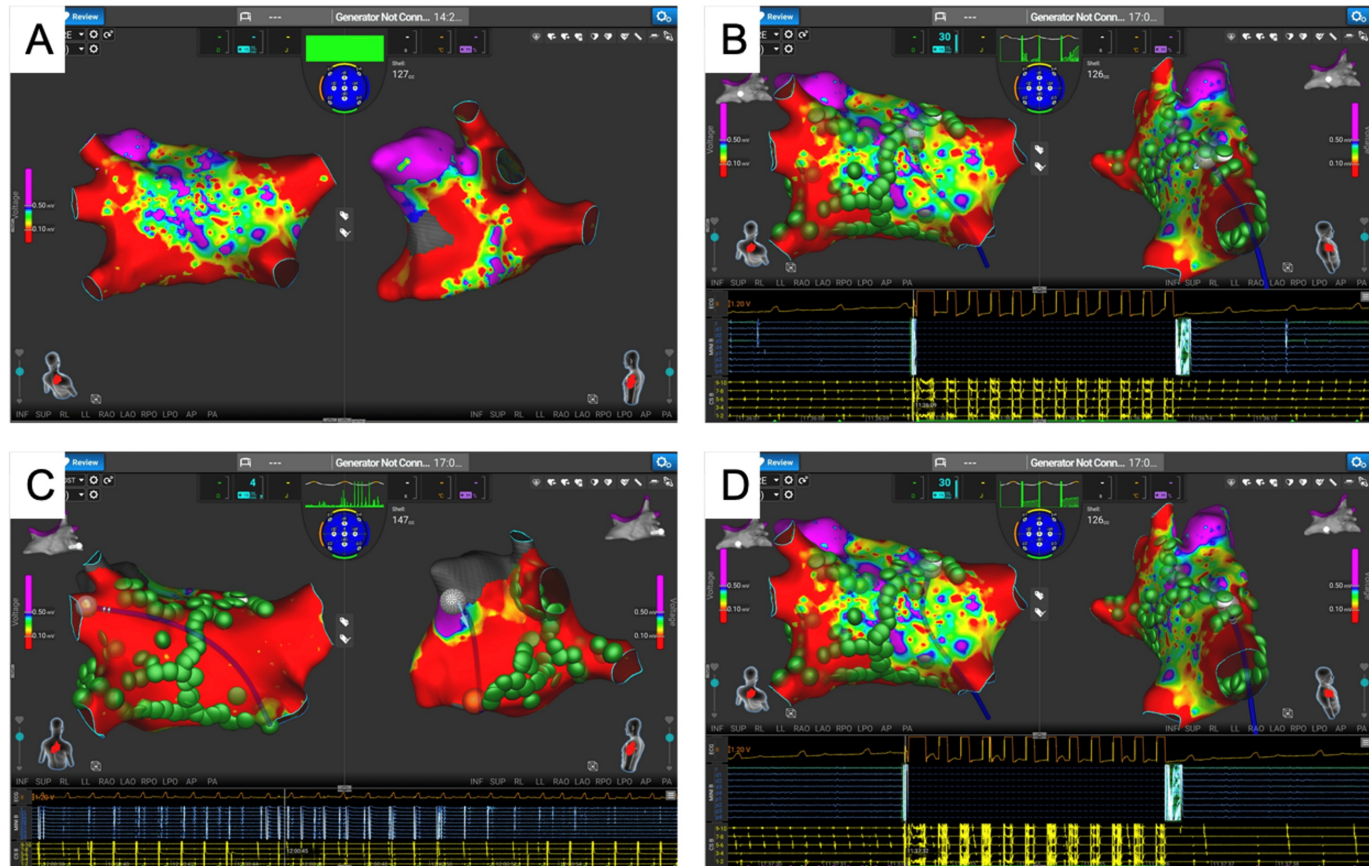
Sequential electroanatomic mapping demonstrating organization and termination of atrial arrhythmia (A) Baseline left atrial bipolar voltage map, demonstrating low-voltage regions consistent with advanced atrial remodeling (voltage scale: bipolar amplitude, mV). (B) Electroanatomic activation map obtained after organization of atrial fibrillation into an atypical atrial flutter during posterior wall ablation, demonstrating changing activation patterns. (C) Left atrial appendage pacing, demonstrating concentric CS activation, confirming bidirectional block across the mitral isthmus. (D) Termination of the organized atrial flutter to normal sinus rhythm following completion of posterior wall and linear ablation, confirmed by intracardiac electrograms CS: coronary sinus

High-resolution electroanatomic mapping using the Medtronic Affera Sphere-9 lattice-tip catheter (Medtronic, Inc., Mounds View, MN) demonstrated a markedly enlarged left atrium with low-voltage regions along the posterior wall, consistent with advanced atrial remodeling (Figure 1A). All four PVs were isolated using PFA. RF energy was applied selectively for lesion consolidation in anatomically challenging regions, including the lateral mitral annulus, rather than as a primary ablation modality.

**Figure 2:**
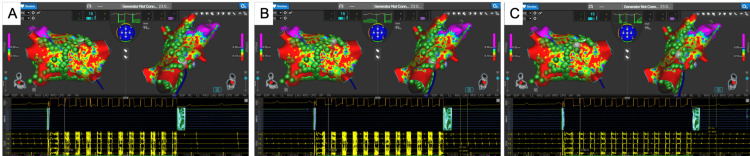
Intracardiac electrograms recorded during catheter ablation (A) Electrograms demonstrating organization of atrial fibrillation into an atypical atrial flutter, with regularized atrial activation and stable cycle length. (B) Electrograms during CTI pacing before completion of the ablation line, demonstrating persistent conduction across the isthmus. (C) Electrograms following completion of CTI ablation, demonstrating bidirectional conduction block, confirmed by differential pacing maneuvers CTI: cavotricuspid isthmus

Despite a successful PVI, the patient remained in AF. During posterior wall ablation, intracardiac electrograms demonstrated progressive organization of AF into an atypical atrial flutter, with increasing cycle length regularity and stable activation patterns, prompting activation mapping (Figures 1B, 2A). Electroanatomic mapping with activation timing overlays demonstrated concentric activation of the coronary sinus (CS), consistent with an organized macroreentrant rhythm. Left atrial appendage pacing further demonstrated concentric CS activation, confirming bidirectional block across the mitral isthmus (Figure 1C).

**Figure 3:**
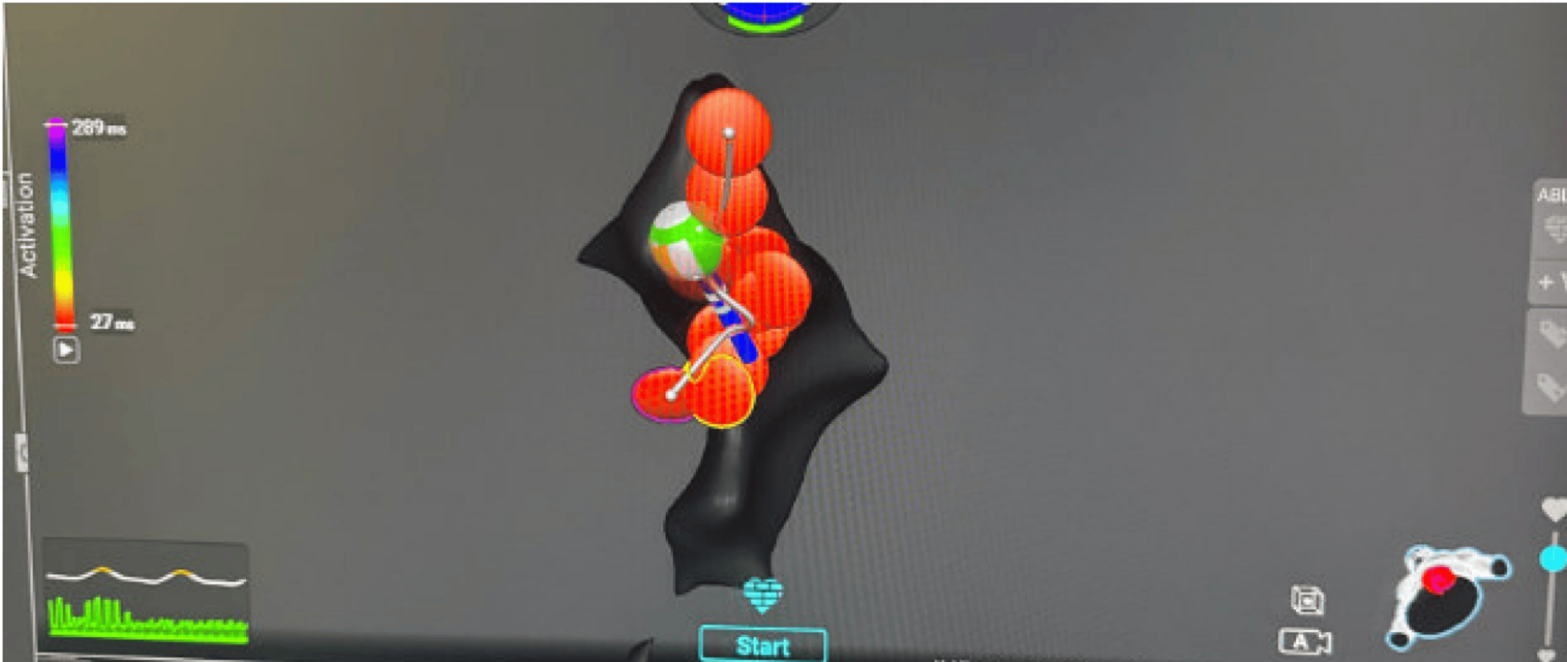
Radiofrequency ablation at the cavotricuspid isthmus Electroanatomic map demonstrating targeted radiofrequency lesion delivery along the cavotricuspid isthmus to achieve bidirectional conduction block

Further ablation resulted in termination of the organized flutter to normal sinus rhythm, confirmed by intracardiac electrograms demonstrating restoration of AV synchrony (Figures 1D, 2A). Given the presence of an organized flutter during lesion creation, additional ablation across the mitral isthmus and cavotricuspid isthmus (CTI) was performed to address the observed macroreentrant substrate rather than as a prophylactic strategy. RF lesions were delivered along the CTI to achieve bidirectional conduction block (Figure 3). Differential pacing maneuvers and intracardiac electrograms demonstrated conduction across the CTI prior to completion of the ablation line and confirmed bidirectional CTI block following lesion delivery (Figures 2B, 2C). No pharmacologic provocation with isoproterenol was performed following completion of the lesion sets.

Postprocedure

**Figure 4:**
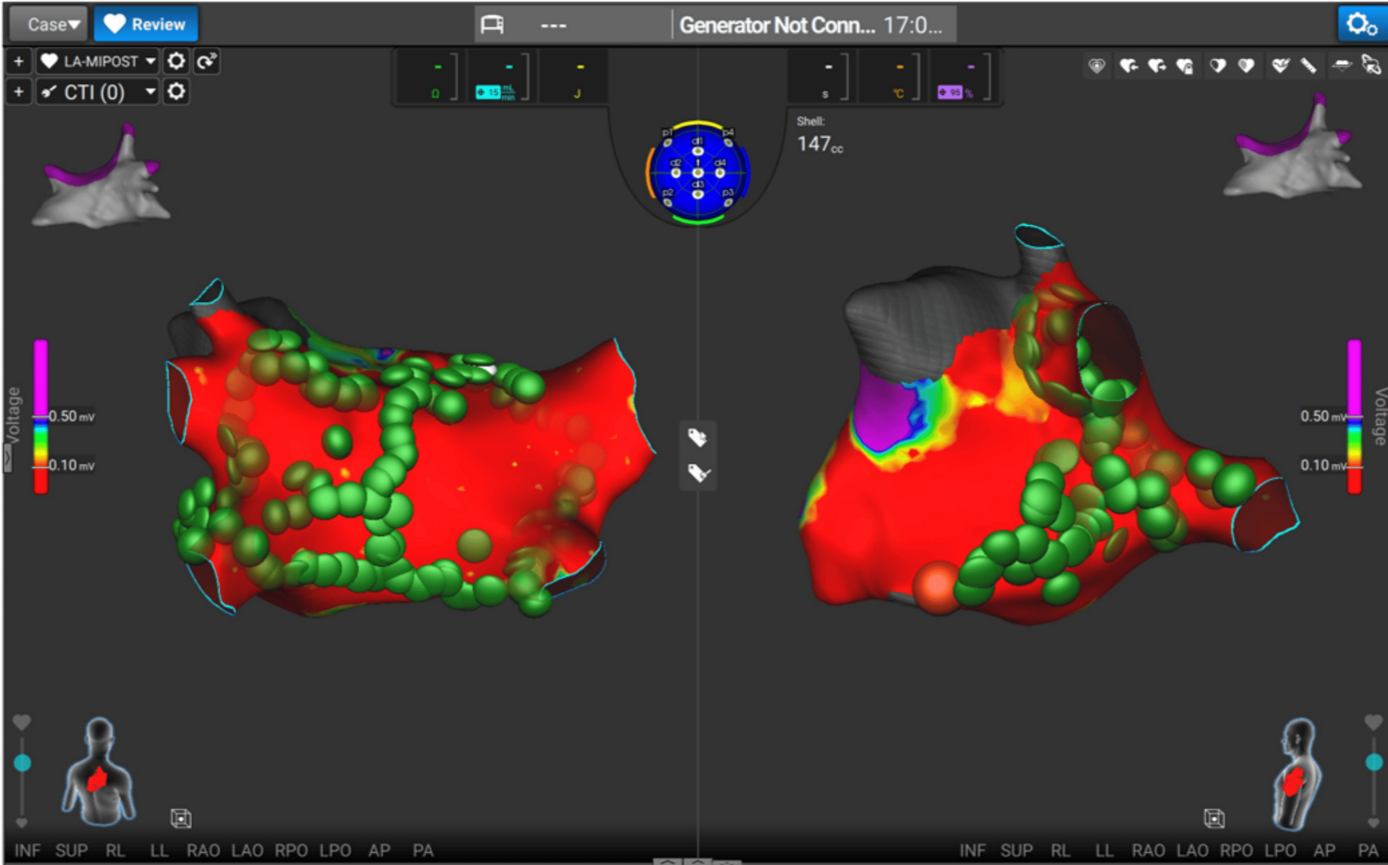
Postablation voltage map, demonstrating durable linear lesions across the mitral isthmus with preservation of healthy atrial myocardium

ICE demonstrated no pericardial effusion, and no phrenic nerve capture or esophageal injury was observed. Postablation electroanatomic voltage mapping demonstrated durable linear lesions across the mitral isthmus with preservation of surrounding healthy atrial myocardium (Figure 4). Intracardiac electrograms obtained at the conclusion of the procedure demonstrated a stable sinus rhythm without inducible atrial arrhythmia. The patient’s sick sinus syndrome did not require temporary or permanent pacing.

## Discussion

This case demonstrates the feasibility of a comprehensive, substrate-based ablation strategy using a focal lattice-tip catheter capable of delivering both PFA and RF energy in a patient with longstanding persistent AF and advanced atrial remodeling. Rather than replicating prior ablation studies, this report contributes to the limited clinical experience with focal lattice-tip PFA for engaging non-PV substrates in complex atrial disease, where outcomes with PVI alone are often suboptimal [3,4]. In this procedure, PFA served as the primary energy source, with RF applied selectively for focal lesion consolidation, reflecting an operator-directed strategy rather than a parallel dual-energy approach.

The patient’s AF was resistant to antiarrhythmic therapy and associated with significant left atrial enlargement, both of which are linked to reduced success of PVI alone [3,4]. Termination of AF during posterior wall PFA suggests involvement of non-PV substrates in arrhythmia maintenance, although this observation should be interpreted cautiously. Intraprocedural rhythm conversion may reflect cumulative lesion burden or progressive atrial organization rather than elimination of a single dominant focus and therefore does not establish causality.

Randomized trials such as STAR-AF II demonstrated no incremental benefit of empiric linear ablation beyond PVI [3]. These trials were conducted using earlier mapping technologies and thermal energy sources, which may not fully reflect contemporary substrate-guided approaches or the tissue-selective properties of PFA. In the present case, additional mitral isthmus and CTI ablation was performed in response to an organized flutter observed during lesion creation, rather than as a strategy to reduce future arrhythmia burden. This approach reflects individualized operator decision-making rather than the evidence-based superiority of extended lesion sets.

Focal lattice-tip catheters allow high-resolution mapping and targeted energy delivery, with the ability to alternate between PFA and RF. This hybrid capability may be advantageous in anatomically complex regions such as the posterior wall or mitral isthmus, although comparative outcome data remain limited. In this case, bidirectional block across all intended lesion sets was confirmed using differential pacing and electrogram assessment, addressing potential macroreentrant circuits associated with arrhythmia persistence and postablation atrial tachycardias [11].

PFA provides a nonthermal ablation mechanism with relative sparing of adjacent structures such as the esophagus and phrenic nerve [6,7]. Large observational studies and registry data have demonstrated a favorable safety profile for PFA, including during posterior wall ablation [12,13], which is particularly relevant in patients with enlarged atria. However, most clinical experience to date involves single-shot or multielectrode PFA systems rather than focal catheter designs.

Contemporary mechanistic analyses emphasize the heterogeneity of AF maintenance in persistent disease and support individualized, substrate-guided ablation strategies rather than uniform lesion sets [14,15]. Within this framework, focal lattice-tip PFA represents a potential tool for selective substrate engagement. Nonetheless, the findings of this report are limited to feasibility and acute procedural success, and the absence of long-term follow-up precludes conclusions regarding durability. Prospective studies evaluating arrhythmia-free survival, durability of linear block, and safety in advanced atrial remodeling are needed.

## Conclusions

Catheter ablation for longstanding persistent AF often requires individualized, substrate-based strategies beyond PVI alone. This case demonstrates the feasibility and acute procedural success of combining PVI with posterior wall, mitral isthmus, and CTI ablation using a focal lattice-tip catheter capable of delivering pulsed-field and RF energy, resulting in intraprocedural restoration of sinus rhythm without cardioversion in a patient with advanced atrial remodeling. The observed termination of AF during posterior wall PFA should be interpreted as a procedural observation rather than evidence of causality, as rhythm conversion may reflect cumulative lesion burden. Accordingly, this single patient report is limited by the absence of long-term follow-up. Future prospective studies are needed to evaluate arrhythmia-free survival, lesion durability, and safety of focal lattice-tip and hybrid PFA/RF strategies in patients with enlarged atria and longstanding persistent AF.
